# Castration of Cryptorchids (Ridglings)

**Published:** 1900-08

**Authors:** V. J. Andre

**Affiliations:** St. Genevieve, Mo.


### CASTRATION OF CRYPTORCHIDS (RIDGLINGS).

By V. J. Andre, V.S.,
ST. GENEVIEVE, MO.

The castration of cryptorchids is an operation which is performed by many without great success. I have seen two cases with this result. In one the horse was cast and held down for four hours, the incision being made on left side of middle line, about six inches long. The hand was introduced by the operator and worked at least an hour, so the farmer said, when the operator, being tired, told his partner to try and locate the testicle; he worked for quite a while, and from one to the other they exchanged turns without success. They told the farmer that the horse must be a clean-cut horse, but the farmer (owner) insisted he was not a clean-cut horse.

As luck would have it, I was coming along the road from a call, and the farmer, knowing me, walked toward the fence and stopped me, saying: "I have two men up there back of the barn trying to alter my ridgling, and they have been there near about four hours and cannot find the testicle. Can you not come in and help them out?" I told him I did not wish to meddle in other peoples' business, as it was not right; but he begged of me to come. I finally consented to go up to the barn. The little I here repeat, as near as I can recollect, is in the farmer's own words.

On the way to the barn, he said: "Those people must be —— quacks, although they said they knew their business, and I consented to let them work on the horse."

On reaching the horse, the owner remarked: "Gentlemen, let this young man try to locate the testicle for you." One remarked: "The h—— with him! He cannot find it any better than we can." The farmer insisted, and said: "Either let him try or let the horse up, as the animal is mine." They finally consented, and said to the owner: "I guess he'll get fooled as we did."

I rolled up my sleeves and immersed my hands in water containing carbolic acid. I made the écraseur ready.

On introducing my hand, I detected at once that the horse was a flanker. Taking the écraseur, in less than four minutes the testicle was detached from the cord—it not being a very large one, about the size of a turkey's egg. The farmer then asked of the other operators: "Where did you learn your business?" One said, "from self-experience;" the other, "from his father."

After the departure of the so-styled castrators, the farmer told me he would always investigate if the party he called to treat or operate on any of his animals was a qualified man or not. If all would do as he said he would do in the future, we would soon stamp out the so-called quackism which is so extensively practised in the United States.

In connection with the little story I have just finished, I will give my method of operating on the cryptorchid.

A horse brought to me for operation is placed in the hospital and kept for twenty-four hours, is given bran-mash, two quarts, at meals, and allowed only one bucket of water at each meal for twenty-four hours; he is then brought out in the yard and examined for hernia, etc., to see if fit for the operation; he is then placed on the mat, the side-lines are placed on the animal, also the trip-rope. When all is ready, I handle the trip-rope, and at same time notify my assistant to pull. When the animal is down, I take it upon myself to tie all ropes, as I am the operator and I must protect myself. The animal being securely bound down, I have a pail brought to the side of him. The pail I commonly use is the milk-bucket, as it is generally the cleanest bucket on the place; get it half-full of rain-water and put in enough creolin to make a solution strength of 1/20. I then use a sponge which I keep in a glass bottle, and use only in castration. I wash the scrotum well and then wring out same sponge and dry off well. My écraseur, which I keep in a chamois sac, lined with white muslin, I place in the creolin solution, having been sterilized before leaving home as well as the other instruments. I then make my incision about one inch to the side of the median line, from before backwards, just long enough to allow the four fingers to go through, only cutting through the skin; then, using the forefinger, break down the fascia, working downward and forward to ring; on reaching the ring I break through just large enough to allow two fingers through. I then insert one hand into rectum, and it is a great assistance in helping to locate the testicle; it is then brought up, and, as near the outer surface as possible, and taken off by the écraseur, which I use in all my ridgling castrations. On entire horses I use the emusculator, which I have found successful in both. It is not necessary for me to give a full description, as my fellow-veterinarian, Dr. Butterfield, of South Montrose, Pa., gave you a very good description in the May number.

After Treatment: Feed lightly; give horse ten miles exercise morning and evening; keep incisions well open, and wash scrotum every morning and evening with lukewarm water and apply weak solution of creolin 1 to 150.

I have operated on eleven, all abdominal, and several flankers. One, a yearling, both testicles in the abdomen; four, three years old; five, six years old; one, ten years old.

One case in particular I wish to relate to you. It was one of the four-year-old; bay horse weighing 1200 pounds, good condition; brought to me to be operated on at the hospital. The following morning he was cast and the operation performed. The testicle taken from this horse weighed nineteen ounces, and in perfectly healthy state. I preserved it in formaldehyde solution, 20 per cent., and will send it to Professor Smith, of the Ontario Veterinary College, to be placed in the museum. This horse was operated on April 19, 1899, with good recovery. I would like to hear from any fellow-veterinarian if he has operated on any cryptorchid and found a larger testicle than the one I have told you about.

I believe the reason that so large a percentage die is that the opening in the peritoneum is made too large and peritonitis sets up, and, furthermore, many veterinarians do not take the necessary antiseptic precautions, thinking, "Oh! well, he is only a horse." Allow me to say we must take the same precautions that the human surgeon does, and try to bring our profession up to a standard as near to that as we possibly can. I think the time will come when the people of this country will look upon us as the people look upon the veterinarian in foreign countries, as England, France, Germany, etc.

